# *QuickStats:* Percentage* of Children and Adolescents Aged 5–17 Years Who Reported Being Tired Most Days or Every Day,^†^ by Age Group and Hours of Screen Time^§^ — National Health Interview Survey, United States, 2020^¶^

**DOI:** 10.15585/mmwr.mm7106a5

**Published:** 2022-02-11

**Figure:**
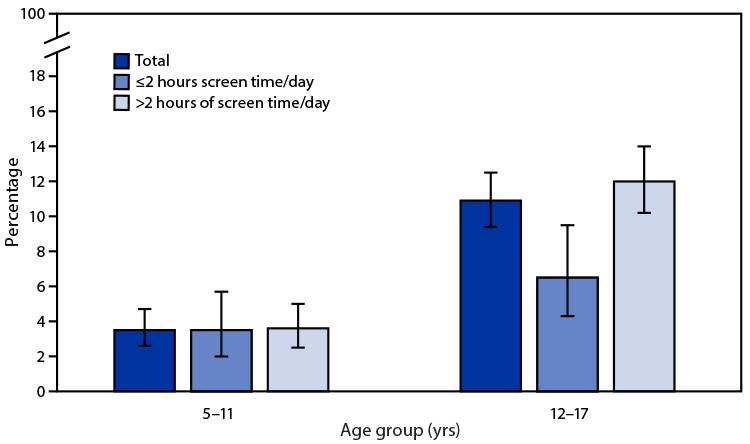
In 2020, 3.5% of children aged 5–11 years and 10.9% of adolescents aged 12–17 years reported being tired on most days or every day. Among adolescents aged 12–17, the percentage reporting being tired was higher (12.0%) for those who reported >2 hours of screen time (in addition to that for schoolwork) per weekday than for those who reported ≤2 hours of screen time each day (6.5%). In children aged 5–11 years, the percentage reporting being tired did not differ by hours of screen time (3.6% for >2 hours versus 3.5% for ≤2 hours). Regardless of the amount of screen time reported, adolescents aged 12–17 years were more likely to report being tired on most days or every day than were children aged 5–11 years.

**Source:** National Center for Health Statistics, National Health Interview Survey, 2020. https://www.cdc.gov/nchs/nhis.htm

